# Consumer Avoidance of Insect Containing Foods: Primary Emotions, Perceptions and Sensory Characteristics Driving Consumers Considerations

**DOI:** 10.3390/foods8080351

**Published:** 2019-08-17

**Authors:** Mauricio Castro, Edgar Chambers

**Affiliations:** Center for Sensory Analysis and Consumer Behavior, Kansas State University, 1310 Research Park Dr., Ice Hall, Manhattan, KS 66502, USA

**Keywords:** insect, food, avoid, disgust, sensory, attitude, psychology, willingness to eat

## Abstract

Why do many human beings find bugs repulsive? Disgust, a psychological factor, is believed to be the main reason why consumers would not consider eating foods containing insect ingredients. This study aimed to understand specific consumers’ behaviors toward insect based products. A global survey was launched in 13 different countries. The participants (*n* = 630 from each country) completed the survey that included demographic questions and questions about why they would or would not eat insect-based products. The results show, particularly for some of the Asian countries, that it is necessary to start exposing and familiarizing the populations about insects in order to diminish the disgust factor associated with insects. It is strongly recommended that an insect-based product should not contain visible insect pieces, which trigger negative associations. The exceptions were consumers in countries such as Mexico and Thailand, evaluated in this study, which did not show significant negative beliefs associated with including insects in their diets. Additional research to promote insect-based product consumption with popular product types might be the first strategy to break the disgust barriers and build acquaintance about insect-based products. The need to educate consumers that not all insects are unhygienic is crucial to eliminating the potentially erroneous concepts from consumer mindsets.

## 1. Introduction

Why do consumers find insects disgusting? By research and definition, disgust is an emotional response of rejection or revulsion to something potentially contagious or something considered offensive, distasteful, or unpleasant [1,2]. Some authors report that it is not the taste that makes food disgusting, but rather the nature and origin of the food that triggers the disgust emotion [3]. Different negative perceptions toward insects, such as being disease transmitters, filthy, unhealthy and unhygienic [4] and the lack of accurate consumer information about insects have built a foundation of disgust. Despite the many excellent reasons to introduce insects to our diets, the current social paradigm is likely to undergo rather drastic alterations before consumers decide to get a side of crickets with their meal or eat other foods containing insect powders as an ingredient [5]. “It’s disgusting” was the main reason, other than not knowing what it is, that consumers said that insect powder was not “natural” [6].

Food perceptions can change, especially if nutritional factors are involved, as reported by Bech-Larsen et al. [7]. In this age of environmental concerns, people are viewing products in new ways. There are other external variables, such as religion and allergic reactions that also can contribute to an increase in rejection to eating insects [5], but disgust has been perceived as the primary motivator. Unfortunately, there is little data showing the actual reasons. Baker et al. [8] suggested that it is necessary to understand more about all the barriers and the food neophobia challenges to fully understand how to reduce the consumer’s negative perceptions and attitudes.

In recent years, different studies have covered the consumer acceptance of insect based products [9], entomophagy [10] and the willingness to eat food produced using insects as an ingredient [11] in one or two countries or regions. However, only one study was found that looked more globally at the issue of insect-based food consumption [5] and that study only examined the issue in terms of willingness to eat such foods and the impact on brand image if companies chose to use such an ingredient. The research helping to understand the actual barriers to insect-based food is minimal. However, Lorenz et al. [12] indicated that the simple fact of contemplating the idea of eating insects provokes an immediate disgust response to the general public. What reasons do consumers from various parts of the world and from different cultures, backgrounds and languages give to eating or not eating insect-based food products? There may be a compendium of thoughts, such as those associated with the consumption of wine (feeling smart and sophisticated), another product that must be learned and is not immediately accepted by most people [13]. If there is only a feeling of fear or disgust preventing people from eating insect-based products, a different challenge is presented than if more extensive concerns must be alleviated.

In consequence, this study aims to understand more thoroughly the psychological and sensory reasons for not eating insects. It does not seek to understand physical or social factors such as allergies or religious restrictions. The study was conducted in 13 countries to provide a somewhat global perspective.

## 2. Materials and Methods

This research was conducted in conjunction with a previously published project [5] where a detailed description of the survey methodology can be found.

### 2.1. Participant Profile

The respondents (*n* = 630 per country) were recruited from existing databases by Qualtrics, one of the world’s largest on-line survey companies. The company’s and its contractor databases include more than 30 million people. Approximately 100 participants of each gender (male, female) and age (18–34 years old; 35–54 years old; 55+ years old) combination completed the questionnaire per country (630 participants per country). A total of 7560 consumers completed the questionnaire.

The participants were from 13 countries (United States (USA), Mexico, Peru, Brazil, United Kingdom (UK), Spain, Russia, India, China, Thailand, Japan, South Africa, and Australia). The differences in the cultures, languages, traditions and religions make this a broad-based multi cross-cultural international survey. However, two additional countries, Egypt and Ghana, were eliminated from the study because not enough participants in each category completed the survey (for all categories in Ghana and for the 55+ age group in Egypt) after multiple attempts. This shows that there were not enough willing consumers with access to an appropriate mobile device to complete the survey in those countries, a limitation of this type of research.

### 2.2. Survey

The global willingness to eat insect products research study was divided in phases [5]. This portion of the survey focused only on the phase that covered the psychological and sensory reasons for not eating insect-based products. The participants indicated their agreement/disagreement on each of the reasons (Table 1) using a 7-point Likert-type scale with 7 as strongly agree to 1, strongly disagree.

The survey was developed based on interviews and focus groups with participants from various countries and many prior studies that identified various issues associated with eating new food products. The final statements were developed based on the aggregate information provided in those focus groups, which was grouped into key topic areas. This portion of the survey focused exclusively on psychological or attitudinal reasons why a person might not eat such products. The English version of the survey was tested for face-validity using four professionals with an understanding of sensory and consumer behavior. The survey was tested for the correctness, use, and timing by seven students of various backgrounds and was pre-tested again using 50 consumers whose data were checked and analyzed to ensure the questions were understandable and did not lead to answers that were inappropriate or unreasonable. 

For statistical modeling (regression analysis), a question on willingness to eat a food product that included an insect-based ingredient was used from Castro and Chambers [5]. The question read: “If a major worldwide company; e.g., Nestle, Coca-Cola, KFC, Starbucks, etc., introduces a new product similar to one you currently buy that contains insect powder, how willing would you be to try this product?”. The number of consumers who were “willing to try”, “not willing to try”, or “not sure” is given in Table 2.

The survey was translated into nine languages (English, Spanish, Portuguese, Russian, Hindi, Mandarin Chinese, Thai, Japanese, and Afrikaans). The inspection of the translations was either by back translation or multiple translation, both with discussion afterwards by the translators to resolve any problems. The single translated versions were offered in some countries (e.g., Russia, UK), but multiple translations appropriate for the country were offered in others (e.g., South Africa, India).

### 2.3. Data Analysis

For each country, the data initially were simply categorized and described using percentages for each potential answer. The next step was to combine the three disagree scores into a category of “disagree” and the same procedure was implemented to the three agree choices obtaining the “agree” category. Score “4”, neither agree nor disagree remained a separate category.

The statistical analyses using multiple regression with a stepwise elimination were performed using MiniTab-18 (Minitab Inc. State College, PA, USA) and SAS 9.4 (SAS Institute Inc., Cary, NC, USA) to estimate the impact of the reasons for not eating products containing insect powder over the dependent variable (willingness to eat food containing insect powder). For every country, the following regression equation was executed:

Y (*Willing to eat products made with insect powder*) = β_o_ + β_1_ Idea Disgusting + β_2_ Taste Not Good + β_3_ Insects Not Safe to Eat + β_4_ Bad Texture + β_5_ Thought makes me Sick + β_6_ Insects are Dirty/Filthy to + β_7_ Color Not Good + β_8_ No Insect Pieces in my Food + ε.

For all the countries, the significant coefficients were described in a bar graph for a better interpretation. Additionally, *r*-squared and *p*-value summaries were noted to understand the percentage of the variation by the models.

This study was approved by the Committee on Research with Human Subjects at Kansas State University.

## 3. Results and Discussion

### 3.1. Psychological/Sensory Reasons for Not Eating Insects Consumers Who Are Unwilling to Eat Insect Powder—Based Products

**Figure 1:**
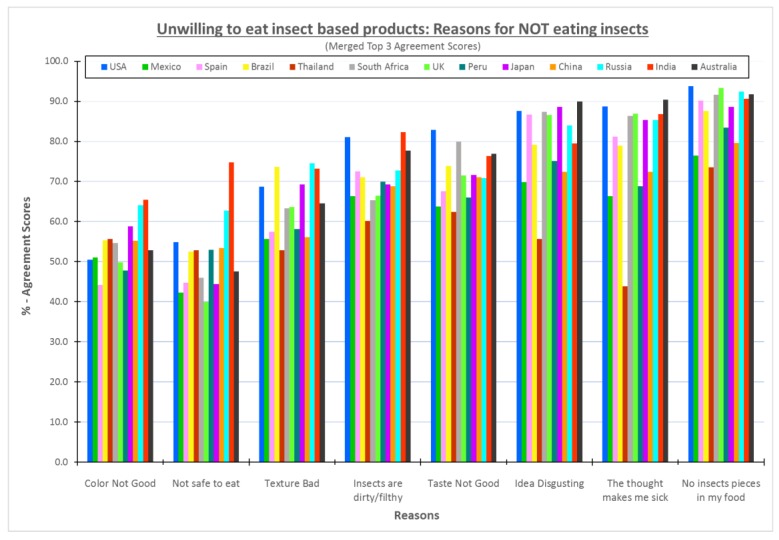
Graph of the reasons for not eating insect based products—Consumers unwilling to try.

Figure 1 shows the percentages of the consumers who were unwilling to try insect powder-based products in each country who selected each psychological or sensory reason for not eating those products. The consumers in all thirteen countries agreed that the most important reason is related to appearance, consumers do not want to see insect pieces in their food, followed by the “Idea is disgusting” or “The thought makes me sick”. The two least important reasons were “The color would not be good” and “Insects are not safe to eat”, although those reasons still averaged approximately 50% of consumers. More than 70% of the participants from each country agreed that appearance is extremely important. It is not appealing to consumers to see insect pieces in their food or snacks. Similar information on the visual appearance was noted by Meyer-Rochow and Hakko [14] who commented that using powders or pastes where the insect was not obvious was a better way to introduce the use of insects in food. 

The primarily English-speaking countries (i.e., USA, Australia and United Kingdom) and South Africa generally were the top countries whose consumers strongly agreed that the reasons for not eating insects were “I do not want to see insect pieces in my food” and “Just the thought makes me sick”/“Idea is disgusting”. Although the number of people who would not eat an insect-based product was high in India to begin with (>65%), with many saying they would not eat such foods based on religious constraints [5], Indian consumers also selected all eight psychological and sensory reasons for not eating insect-based products at a high percentages (65–90%). This probably is because the concept of eating a meat-containing food is anathema to most Hindus and any reason would be a reason not to eat such foods. Mexican and Thai consumers were the least likely to reject an insect-based product [5], but those consumers who said they would not try such a product did not agree on reasons for not eating insect-based foods. The percentage of consumers in Mexico and Thailand who chose particular reasons for not eating insect-based foods were among the lowest of all countries for all reasons except the color. Fu and others [15] showed that cultural values modulate consumer beliefs. Therefore, this study hypothesized that when a product is acceptable to the culture (i.e., more people are willing to eat than not eat that food), it is likely that individual beliefs related to food become the key decision criteria. In the case of Mexico and Thailand, it is likely that the reasons for not eating a food are more individualized and, thus, more likely to be spread among many factors than when overarching cultural or societal norms are present. 

### 3.2. Consumers Who Are Willing to Eat Insect Based Products

**Figure 2:**
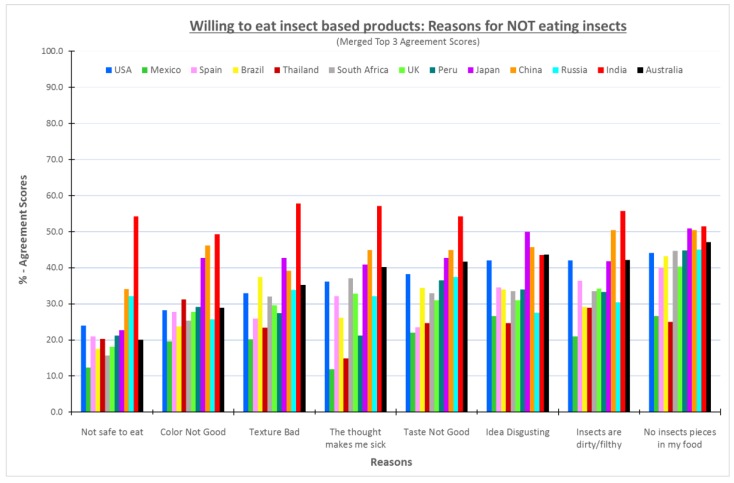
A graph of the reasons for not eating insect based products—consumers willing to try.

Figure 2 shows the percentages of the consumers who were willing to try insect products in each country who selected each reason for not eating an insect powder-based food product. Not surprisingly, the results showed that for most countries except India, the consumers who were willing to try insect-based foods generally did not have reasons for not choosing such foods. In most cases, less than 40% of consumers in those countries chose a specific reason for not choosing an insect-based food product. There were two major exceptions. More than 40% of consumers willing to try insect-based product in most countries indicated they still would not choose a food if there were insect pieces in it. Second, even those Indian consumers who were willing to try insect-based products found many reasons not to eat such products. Almost every reason, except disgust, was chosen by 50% or more of willing Indian consumers as a reason not to eat insect-based products. It is not surprising that even those who say they are willing to try a food, find reasons for not eating such foods. From other research [16,17,18,19], it is known that liking and pleasure, habits, convenience, hunger, health, and a myriad of other reasons motivate people to eat the foods they choose. 

When considering the reasons for willing consumers, the same patterns overall were observed in reasons as the unwilling consumers. The main reason for not eating such products being the appearance of pieces. Mexico and Thailand rated the lowest agreement scores across all the reasons. Over 40% of Chinese and Japanese consumers agreed to the following reasons: Color not good, the thought makes me sick, taste not good, the idea is disgusting, insects are dirty/filthy, and no insect pieces in my food. The results were expected to show low agreement scores because these consumers were willing to eat food obtained from insects. However, it is interesting that the pattern of responses is similar to those from unwilling consumers. This information suggests that the decision to eat or not eat a particular food made from insects has the same barriers regardless of the individuals’ willingness to try such products. This is good news for those who wish to promote the use of insects as a food ingredient because some types of information provided to consumers have been found to increase likelihood to eat insect-based products [20,21].

### 3.3. Regression Analysis—Reasons

**Figure 3:**
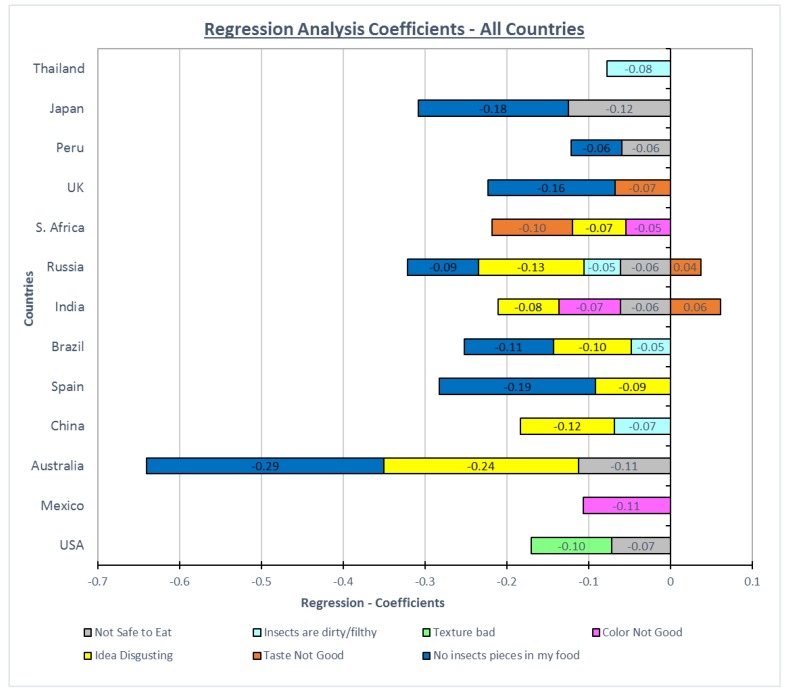
Regression analysis coefficients—the stepwise method.

The participants’ responses from all countries showed similarity in which responses were less important to them. Before conducting the multiple linear regression analysis, a covariance was detected between two of the reasons. The idea is disgusting and the thought makes me sick were highly correlated and therefore the “thought makes me sick” was dropped from the models because the term “disgust” was commonly used to describe this emotional construct related to insects [12]. Figure 3 shows the regression coefficients for the seven reasons for not considering eating foods with insect-based components as an ingredient. 

“The texture would be bad”, was removed from further analysis during the multilinear regression analysis (stepwise procedure) because this variable was not significant and, ultimately, was eliminated from the equation in all but one country (USA). The remaining six reasons were compared and analyzed with the appearance and disgust being the two independent variables that were present in most of the countries’ regression equations. Thus, consumers emphasized a sensory factor—the visual appearance of “insect pieces”, an emotional factor—disgust, and a psychological belief/trust factor—“insects are not safe to eat”, as primary motivations for not eating insect-based products. Recent research has found similar constructs to be barriers for the consumption of insect-based foods (new FOODS). The rest of the variables, insects are dirty/filthy, taste not good and color, not good were small and generally irrelevant reasons that either were co-dependent on other reasons or did not affect the willingness to eat insect-based food products once other considerations were noted.

### 3.4. Specific Reasons for Not Eating Insect-Based Foods

#### 3.4.1. No Insect Pieces in My Food—All Consumers Considered

**Figure 4:**
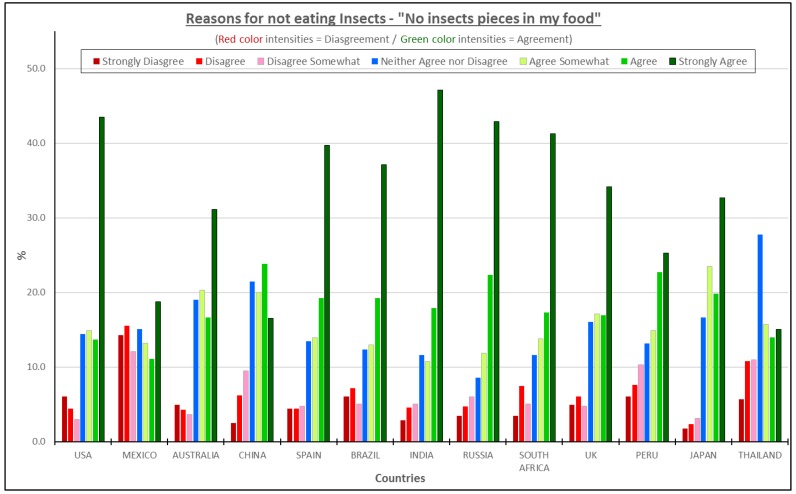
The reasons for not eating insect products—“Do not want insect pieces in my food”.

When consumers were asked for the reasons that they would not consider eating foods containing insect powder as an ingredient, over 60% of the participants in China, Peru, Australia, UK, Brazil, Russia, South Africa, Spain, India, Japan, and USA strongly agree, agree or somewhat agree that the appearance was extremely important and did not want insect pieces in the food. In Mexico and Thailand, the percentages were also considered high. Over 40% of the respondents agreed with the statement, “no insect pieces in my food” (Figure 4).

In most of the countries except for South Africa (*ρ* = 0.38) and Thailand (*ρ* = 0.35), the reason “I do not want insect pieces in my food” was highly correlated to “The idea is disgusting” with correlation scores over 0.50. When consumers can see insects or pieces in their food, the food becomes more disgusting. This is a visual sensory cue that should be easy to correct (not seeing insect pieces in my food) by grinding the insects into a powder (flour). Grinding would avoid any visual parts of the insects in the food to be prepared. During the regression analysis, the texture reason was eliminated which confirms that consumers were focusing more into the appearance/visual aspect of the product.

#### 3.4.2. The Idea Is Disgusting—All Consumers Considered

**Figure 5:**
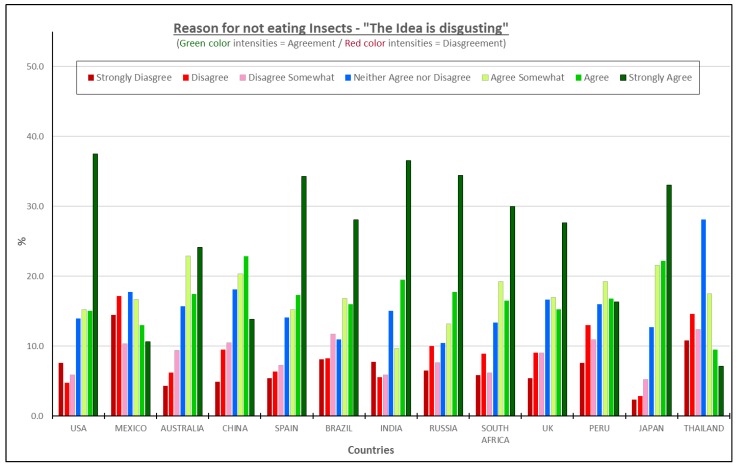
The reasons for not eating insect products—“The idea is disgusting”.

More than 50% of the participants in each of the countries, except for Mexico and Thailand, shared the idea that using insects as an ingredient in food is disgusting. Japanese consumers scored this reason the highest out of the six reasons and the highest of all the thirteen countries, that is, 77% of the respondents agree that the idea of eating insect-based products is disgusting. The USA (68%) and Spain (67%) completed the top three countries for this concept (Figure 5).

The high percentages for disgust align with the regression coefficients showing that the disgust factor is the second most significant reason for not eating insects after the reason “No insect pieces in my food”. Therefore, for such products to be successful, it is essential to begin breaking this emotional barrier by developing insect-based products that are familiar to consumers to show that insects can simply be another ingredient as opposed to a contaminant. 

There are many foods whose ingredients alone are not appealing to consumers either because they may not be natural, organic, GMO free, etc., [6,22] but may not cause the same level of emotional response in an actual food product. The more exposure to these kinds of typical products made with insect ingredients and education about the benefits of insects as food, the more probability there is to decrease the disgust factor.

#### 3.4.3. Insects Are Not Safe—All Consumers Considered

**Figure 6:**
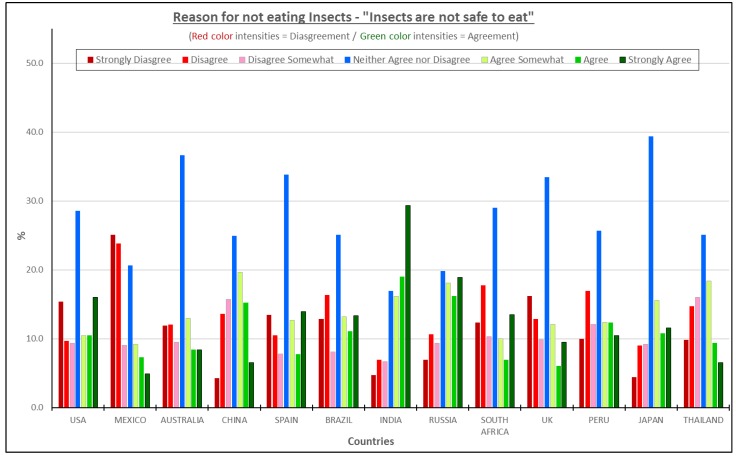
The reasons for not eating insect products—“Insects are not safe to eat”.

For all the countries, except for Mexico and India, the statement “Insects are not safe to eat” was largely considered to be a neutral statement (Figure 6), although it appeared in some regression coefficients as a negative factor (Figure 3). Most consumers in those countries scored it neither, agree nor disagree. However, 65% of consumers in India agreed to this concept, while in Mexico barely 20% disagreed with that statement. The uncertainty about the safety of insects is a topic that needs further research both from the standpoint of how it impacts the potential use of insect-based foods and the human health perspective. Castro et al. [5] recently reported that people believe insects carry diseases and some people believe themselves to be allergic to insects. Those are powerful reasons to question the safety of insect-based foods for those consumers. Studies have identified that consumers might experience similar allergic reactions to seafood when insects are consumed [23].

Furthermore, research in conjunction with clinical studies is necessary from the human standpoint to prove which specific diseases insects might transmit to humans and what chemical components and parts of the insects could provoke allergic reactions [24]. In addition, conclusive zoonotic diseases need extensive research to diminish the concept that “Insects are not safe”.

#### 3.4.4. Insects Are Dirty/Filthy—All Consumers Considered

**Figure 7:**
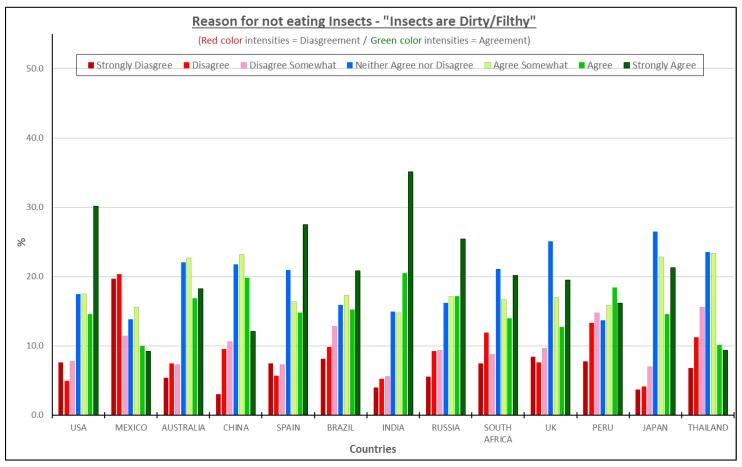
The reasons for not eating insect products—“Insects are dirty/filthy”.

The percentages of consumers who agreed that insects are dirty-filthy trends lower than the first three concepts discussed (Figure 7). India, USA and Japan, are the top three countries associating insects with filth or dirt. 

For those three countries, the percentages of consumers, ranged from approximately 60–70%. Less than 35% of Mexican consumers agreed with that statement, the lowest of all the countries. Peru and Thailand round out the three countries with the lowest agreement on this statement. This reason might be associated with previous perceptions or misconceptions about intoxicating bacteria, viruses, and parasites or the fact that some insects are connected to waste or decay material [25].

#### 3.4.5. Taste Not Good—All Consumers Considered

**Figure 8:**
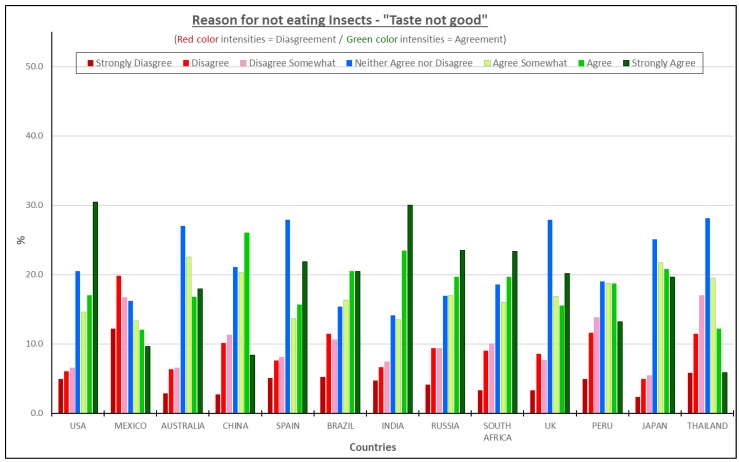
Reasons for not eating insect products—“I do not think it would taste good”.

The consumers’ responses to the agreement “I do not think it would taste good” (Figure 8) resemble results from the reason “Insects are dirty/filthy” (Figure 7). India, Japan, USA, and Russia are the countries with the highest percentage of people agreeing with this reason, all slightly higher than 60%. Mexico and Thailand showed the lowest percentage of people agreeing with that statement. Mexico was the only country where the disagreement response was higher than the agreement rate. It is important to highlight that “Taste not good” is the highest reason related to ingested sensory properties although the visual perception of insects or insect pieces may imply ingested effects. One caution is that a number of consumers choose “neither agree nor disagree”, suggesting that consumers are not sure of how insect-based products would taste and are not confident that an insect based product would taste good. This may suggest that insects are not a barrier from an ingested sensory standpoint, per se, but that the visual perception of insects promotes associations with disgust or textures that would not be desirable. This is a great opportunity to conduct sensory discrimination tests to evaluate if consumers can differentiate between a regular product and an insect product and if they can to conduct descriptive sensory studies to determine the actual sensory differences in those products. 

Several studies focused on the sensory aspect of overall liking to determine the consumer’s behavior towards insect products [26,27,28]. The conclusions from those studies showed that the participants’ overall liking was influenced by the appearance and taste. In addition, it was noted in some studies that the insect parts needed to be invisible for acceptance, which is similar to the findings related to appearance considerations in this study. In a small study with 26 students who were blindfolded and held their noses, slightly more than half could identify the processed insect samples from cheese, dried fish or bread [14]. The authors concluded that this indicated potential acceptance if consumers ate products containing insect flours or pastes where the consumers could not see the insects. In one study [26] of burgers, men rated the insect burger between the beef and lentil burger, with a preference for the mealworm and beef burger. It should be noted in all the prior studies, only those consumers who indicated they were willing and interested in eating insect-based products were used, which may skew results more positively.

#### 3.4.6. Color Would Not Be Good—All Consumers Considered

**Figure 9:**
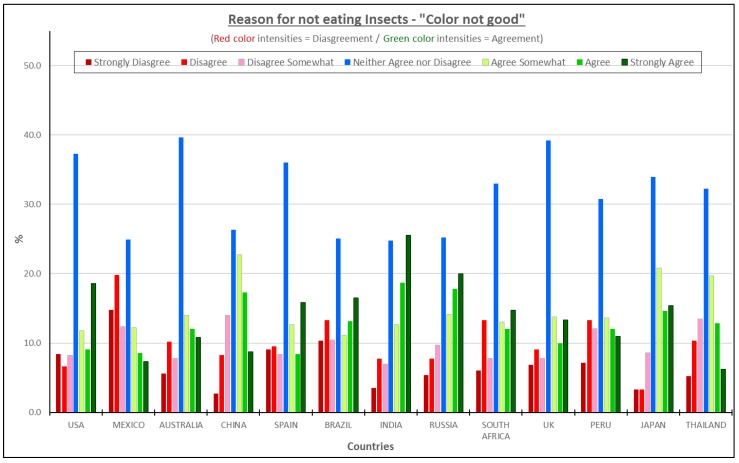
The reasons for not eating insect products—“Color would not be good”.

A further sensory consideration is color. Over 50% of the participants in India, Russia and Japan agreed that the color of an insect based product would not be good (Figure 9). Interestingly, the Latin American countries (Mexico, Peru, and Brazil) were the only countries where more or almost more consumers disagreed with that statement than agreed. It must be noted that this statement generated uncertainty (high percentages of neither agree nor disagree) similar to the statement “Insects are not safe to eat”, suggesting that consumers simply were not sure what the impact of insect ingredients would be on color. Of course, if insect ingredients do result in a color issue, that color might be altered or fixed by adding natural and familiar colors to imitate the original color of a specific product category.

## 4. Conclusions

This study provides a better understanding of the reasons that consumers would not consider eating foods containing insect ingredients. The appearance is a critical issue and a high priority for consumers. There is no doubt that the fragments or pieces of the insect cannot be present in the final product. The emotional and psychological issues represented by the statements “The idea is disgusting”/“Just the thought makes me sick” and the potential misconception that all “insects are not safe to eat” are as crucial as the visual factor.

Of lesser impact in this study, but potentially related to the key factors are such aspects as the misbelief that all insects are dirty/filthy, which may cause consumers to avoid insect-based products. The two sensory characteristics that concerned some participants were the potential impact of the insect ingredients on the taste and texture, but those should be able to be overcome with the adequate selection and formulation of the food product. Certainly, those two aspects are barriers for any new or revamped products that research and development groups need to carefully consider before the creation of a new product.

This research suggests that the use of insect-based powders/flours to avoid the appearance and textural issues and education that overcomes the disgust and safety concerns of consumers are key to the introduction of insect-based food products in many countries. In some countries, such as Mexico and Thailand, the sensory issues may be of more concern than the disgust issues because insect-based products are already known, although not necessarily widely eaten.

## Figures and Tables

**Table 1:** The reasons for not eating foods containing insect powder as an ingredient.

Reasons for not Eating Insect-Based Products Scale (7-Point Likert Type)
1. The idea is disgusting
2. I do not think it would taste good
3. Insects are not safe to eat
4. The texture would be bad
5. Just the thought makes me sick
6. Insects are dirty/filthy
7. Color would not be good
8. I do not want insect pieces in my foods

Scale: 1 = Strongly disagree, 2 = Disagree, 3 = Somewhat disagree, 4 = Neither agree nor disagree, 5 = Somewhat agree, 6 = agree, 7 = Strongly agree.

**Table 2:** The number (*n*) of consumers in each country who were not, were, and not sure about eating an insect-based product from a well-known manufacturer (total *n* = 630/country; sorted by not willing).

Country	Not Willing, *n*	Willing, *n*	Not Sure, *n*
Russia	399	203	28
Japan	390	131	109
India	364	205	61
Spain	356	205	69
USA	324	218	88
South Africa	311	248	71
England	307	227	96
Australia	294	216	120
Brazil	278	282	70
China	216	278	136
Peru	203	363	64
Thailand	186	353	91
Mexico	131	450	49
